# Ten-year survival of ceramic-on-ceramic total hip arthroplasty in patients younger than 60 years: a systematic review and meta-analysis

**DOI:** 10.1186/s13018-021-02828-1

**Published:** 2021-11-18

**Authors:** Ihab Ibraheam El-Desouky, Albaraa Hassan Helal, Ali Mohamed Reda Mansour

**Affiliations:** 1 Faculty of Medicine, Kasr Alainy School of Medicine, Cairo University, Cairo, Egypt; 2 Faculty of Medicine, 6th Oct. University, Giza, Egypt

**Keywords:** Ceramic, Arthroplasty, Hip, Non-elderly

## Abstract

**Background:**

Total hip arthroplasty (THA) with ceramic-on-ceramic (CoC) was created to minimise wear debris and aseptic loosening. A decade ago, a meta-analysis showed a 10-year survival rate of just 89%. Based on the excellent tribology of the current CoC, significant improvement of implant survivorship is expected. In patients younger than 60, we conducted a meta-analysis to assess 10-year survival and complications after using current primary CoC THA.

**Materials and methods:**

PubMed, Scopus, EMBASE, Virtual Health Library, and Cochrane Library were used to scan for published trials that met the inclusion criteria until January 2019. The qualified studies were subjected to a systematic review and proportional analysis, and the randomised controlled trials (RCTs) were included in a comparison meta-analysis.

**Results:**

Thirteen studies were included 156 findings. The total number of hips was 2278. Nine studies were cohort, and four were RCTs between ceramic and polyethylene cups. The analysis revealed an average age of 44 years (range 24–54). The 10-year survival 96% (95% CI; 95.4–96.8%), aseptic loosening rate 0.516. (95% CI; 0.265–0.903), ceramic fracture rate 0.620 (95% CI; 0.34–1.034) and squeaking rate 2.687 (95% CI; 1.279–4.593). A comparison meta-analysis revealed the risk ratio (RR) for revision was 0.27 (95% CI; 0.15–0.47), and for aseptic loosening 0.15 (0.03–0.70) favouring CoC, while RR for component fracture was 1.62 (95% CI; 0.27–9.66) favouring the polyethylene.

**Conclusion:**

In patients under sixty, current CoC THAs are correlated with better 10-year outcomes than before and have high survivorship rates.

*Level of evidence*: Level I.

## Background

With longer life expectancy, total hip arthroplasties (THA) in young patients are likely to wear out faster with higher rates of aseptic loosening. While there is agreement on cementless fixation, there is no such agreement on articulating surfaces [1].

Ceramic-on-ceramic (CoC) THAs have low friction and wear due to their mechanical and chemical properties and surface lubrication by joint fluid, resulting in less osteolysis than other bearing surfaces currently available [2, 3].

Ceramics' brittleness, on the other hand, raises the possibility of component fracture. The replaced joint's squeaks are also a cause of concern. In the young and active patient, the best bearing surface for THA is still up for discussion [4–7]. So, continuous research to develop CoC bearings' properties could have more prolonged survival and fewer problems in the 3rd- and 4th-generation ceramic bearings [8].

Thus, we performed a systemic review and meta-analysis of studies that reported 10-year survival based on the CoC bearing of the 3rd-and 4th-generation CoC THAs in patients younger than 60 to address their complications and survival rate.

## Materials and methods

### Search strategy

In Jan 2019, the study was conducted according to Preferred Reporting Items for Systematic Reviews and Meta-Analyses statement (PRISMA) rules [9]. Electronic searches were conducted in PubMed, Scopus, EMBASE, Virtual Health Library, and the Cochrane Library to identify relevant articles.

Medical Subject Headings (MeSH) terms and free words were used, including ceramic (CoC, alumina) and hip arthroplasty (THA, total hip replacement). Two investigators reviewed each article independently to identify the publication relevance to the eligibility criteria included primary THA for patients < 60 years with CoC bearing surfaces of the 3rd- (*Forte*) and 4th-(*Delta*) generations with reported 10-year survival. Studies included near 10-year results or more of Biolox *forte* or *delta*, full text in English*,* prospective or retrospective, cohort or RCTs were included.

Exclusion criteria were animal studies, case reports or studies with less than 15 patients, scientific correspondence, poster, conference, thesis, guidelines, and comments. Studies that included revision surgeries, insufficient data, or unclear identification of the patient population, or used implants were also excluded.

### Data extraction

After eligibility of any study, two investigators independently extracted data from each report that included the first author's family name, year of publication, material design, patient demographic data, enrollment period, follow-up period, type of implant fixation, the bearing surfaces generation, and the outcomes.

### Outcome measures

A proportional analysis for the eligible studies included the primary outcome, i.e. 10-year implant survival, was analysed for the systematic review. The secondary outcomes comprised the aseptic loosening rate, audible squeaks, ceramic component fractures, and the frequency of these complications for each bearing surface generation. A comparison meta-analysis of RCTs was calculated, including the revision rate, aseptic loosening, and component fracture.

### Quality assessment

The quality of cohort studies was assessed by the Newcastle Ottawa Scale (NOS) [10, 11]. It has eight items, collected into three groups: population choice, the comparability of the groups, and the ascertainment of the exposure or outcome. A study can be considered as the highest quality if it is awarded nine stars.

### Statistical analysis

The included studies were pooled using the Mantel–Haenszel fixed-effects method (FEM) and the DerSimonia Laird random-effects method (REM). In the absence of significant heterogeneity, the FEM was considered; otherwise, the REM was considered.

Studies included were tested for heterogeneity using the following tests:

1. Cochran Q chi-square test: A statistically significant test (*p* value < 0.01) denoted heterogeneity among the studies.
2. I-squared (I2) index, which is calculated as follows: $I^{2} = \left( {\frac{Q - df}{Q}} \right)*100\%$ where *Q* is the chi-squared statistic and df is its degrees of freedom.

In the case of heterogeneity >50% at *I*^2^ test, a Mantel-Haenszel random-effect was planned. The continuous outcomes were expressed as mean and standard error (SE) and 95% confidence limits (95% CI). The odds ratio (OR) with 95% CI was calculated for binary outcomes. Data for survival percentage (frequency) were pooled using the inverse-variance method and the Freeman-Tukey double arcsine transformation to calculate proportion before returning to the original scale for plotting.

Statistical analyses of the meta-analysis were performed using RevMan V.5.3 (The Cochrane Collaboration, 2012). A two-sided *p* value < 0.05 denoted statistical significance.

## Results

### Search results

**Fig. 1:**
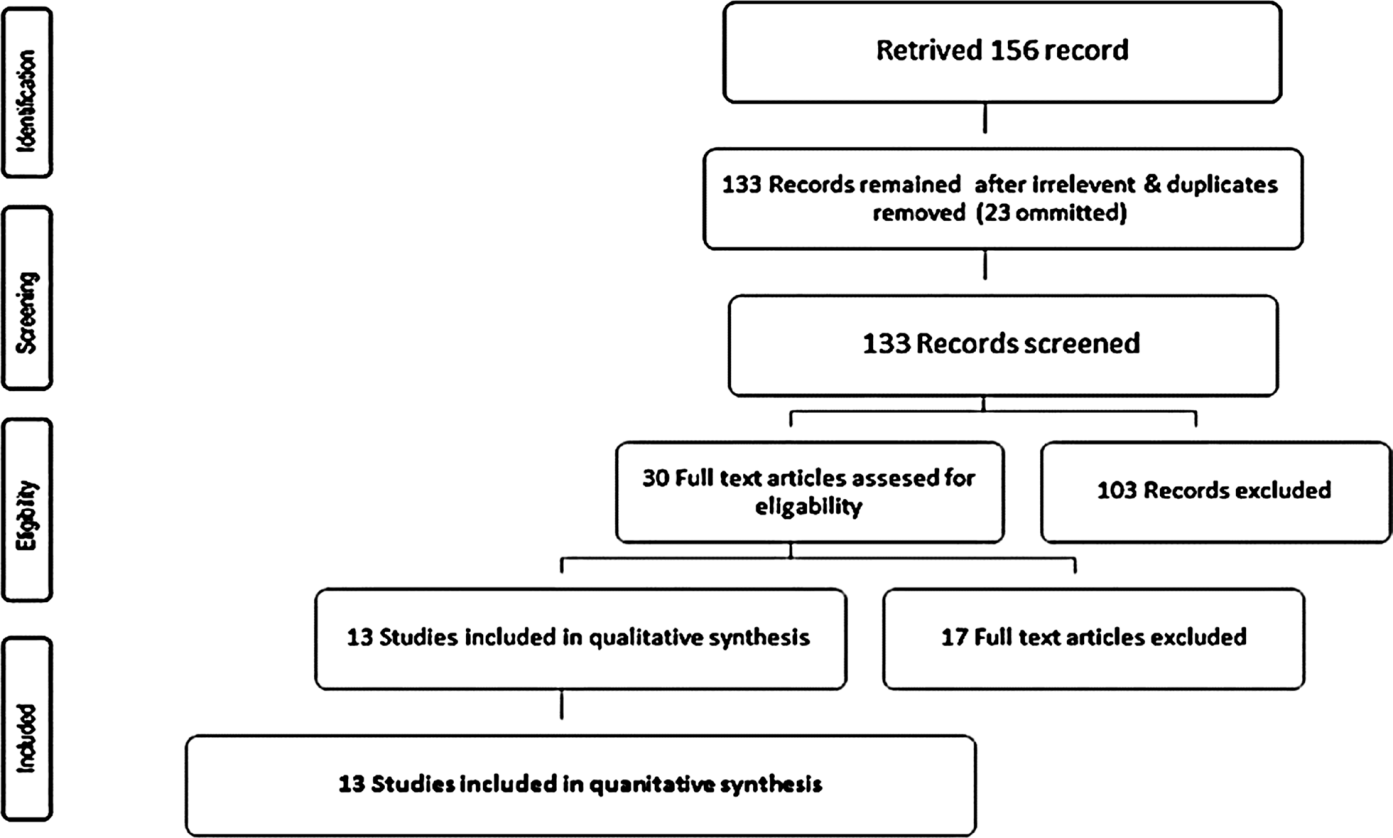
PRISMA flow diagram for selection of studies

We identified a total of 156 studies, 23 duplicates were omitted. After application of our eligibility and exclusion criteria, we excluded 103 records. Due to the low quality of research according to NOS, we again excluded nine records. Again, eight records were excluded: due to the inaccessibility of English full texts. That leaves us with 13 records available for data extraction and quantitative synthesis. Figure 1

These studies included four RCTs and nine cohort studies; five studies were prospective, four studies were retrospective, and all were published in peer-review journals between 2006 and 2018 [6, 12–23]. The total number of the included hips was 2278. The study sample sizes ranged from 29 to 930 patients per the study, with an average age of 44 (24–54 years). The average follow-up was around 10 years in each study (9.7–15 years). Biolox Forte ceramic articulations were used in 1339 hips, while Biolox Delta articulations were used in 939 hips in two studies [17, 22] Table 1.

**Table 1:** Patients and implant characteristics based on the studies

Study	Hips no	age (years) at surgery	Study type	Implant (Bioloox)	Fixation uncemented = both cup and stem	Outcome Score	Aseptic loosening	Prosthetic fracture	Bearing fracture	Squeaking	Follow-up (years)	K-M survival at 10 years (%)
Murphy 2006 [12]	174	50	Prospective	Forte	Uncemented	98	1	1	1	0	10	99.3
Boyer. 2010 [13]	76	39	prospective	Forte	Uncemented cup + cemented stem	96	4	1	1	1	11	92
Lee 2010 [14]	88	41	retrospective	Forte	Uncemented	96	0	2	2	13	10	99
Kim et al. 2010 [15]	93	38	prospective	Forte	Uncemented	96	0	0	0	1	11.1	100
Kress et al. 2011 [16]	62	50	prospective	Forte	Uncemented	90	1	0	0	0	11	99
Mesko et al. 2011 [17]	930	51	RCT	Forte/delta	Uncemented	97	2	27	3	21	10	97
D’Antonio et al. 2012 [18]	189	54	RCT	Forte	Uncemented	96	0	6	1	3	10.3	99.3
Yoon et al. 2012 [19]	75	24	retrospective	Forte	Uncemented	97	0	1	1	12	10	98,9
Beaupre et al. 2016 [20]	48	53	RCT	Forte	Uncemented	98	0	2	0	2	11	94
Wang et al. 2016 [21]	90	40	retrospective	Forte	Uncemented	98	0	2	2	1	9.7	97.3
Kim et al. 2016 [22]	334	48	prospective	Delta	Uncemented	98	2	0	0	5	13.1	99.7
Atrey et al. 2018 [6]	29	41	RCT	Forte	Uncemented	96	0	1	1	0	15	89
Lau et al. 2018 [23]	90	40	retrospective	Forte	Uncemented	39.8/48	1	1	1	0	12.1	96.4

### Quality assessment of bias risk

In general, the methodological quality of all the cohort trials was low in bias risk. In terms of NOS, the mean value was 6.7 stars. Three studies got a 6-star rating [12, 16, 19], five studies got a 7-star rating [13–15, 21, 23] and one study got an 8-star rating [22].

The proportional analysis was conducted for all 13 studies, and the comparison meta-analysis included four RCTs [6, 17, 18, 20] comparing COC-bearing surfaces to the polyethylene group in THA.

### 1-Proportional analysis

**Fig. 2:**
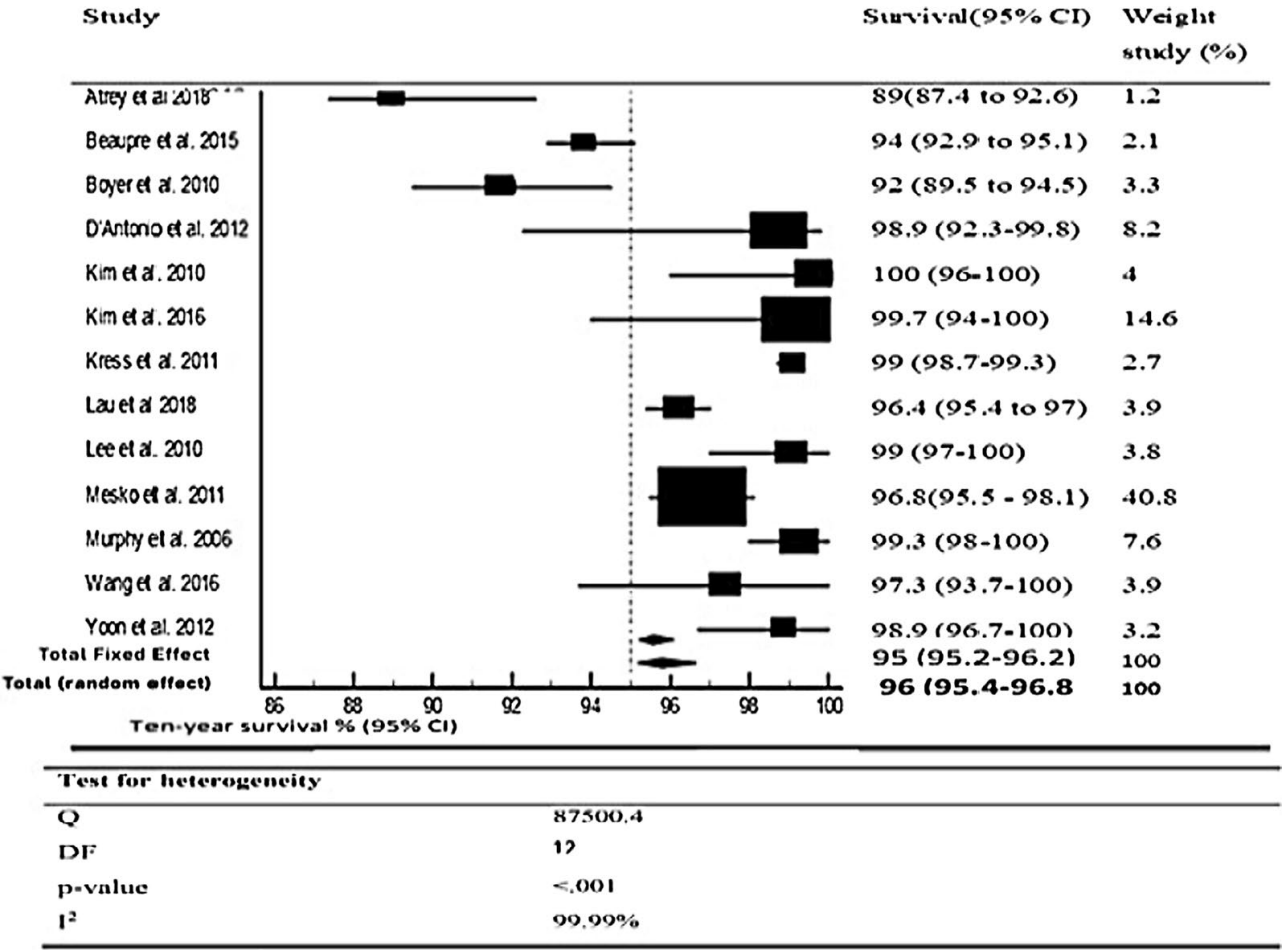
Forest plot for ten-year survivorship

10-year *survival* The mean 10-year survivorship was 96% (95% CI; 95.4–96.8 *p* < 0.001). The random-effects model was used due to considerable heterogeneity (*I*^2^ = 99%). The proportion plot at the end of ten years is shown in Fig. 2.

*Aseptic loosening* rate was 0.516 (95% CI; 0.265–0.903). Table 2 There was un-important heterogeneity (*I*^2^ = 22%); therefore, the fixed-effects model has used.

**Table 2:** Aseptic loosening

Study	Sample size	Proportion (%)	95% CI	Weight (%)	
				Fixed	Random
Atrey 2018	29	0.000	0.000–11.944	1.31	2.04
Beaupre 2015	48	0.000	0.000–7.397	2.14	3.23
Boyer 2010	76	5.263	1.452–12.931	3.36	4.84
D'Antonio 2012	189	0.000	0.000–1.933	8.29	10.07
Kim 2010	93	0.000	0.000–3.889	4.10	5.75
Kim 2016	334	0.599	0.0726–2.146	14.62	14.78
Kress 2011	62	1.613	0.0408–8.662	2.75	4.05
Lau 2018	90	1.111	0.0281–6.036	3.97	5.59
Lee 2010	88	0.000	0.000–4.105	3.88	5.48
Mesko 2011	930	0.215	0.0261–0.775	40.64	24.33
Murphy 2006	174	0.575	0.0145–3.160	7.64	9.47
Wang 2016	90	0.000	0.000–4.016	3.97	5.59
Yoon 2012	75	0.000	0.000–4.800	3.32	4.78
Total (fixed effects)	**2278**	**0.516**	**0.265**–**0.903**	**100.00**	**100.00**
Total (random effects)	2278	0.614	0.271–1.092	100.00	100.00

The bold line indicates that a fixed-effects model was used rather than a random-effects model to account for modest outcome heterogeneity

*The audible squeaking* rate was 2.687% (95% CI; 1.279–4.593). There was considerable heterogeneity (*I*^2^ = 84.6%); therefore, the random-effects model was used.

*Component fracture* rate was 0.62% (95% CI; 0.341–1.034). There was moderate heterogeneity (*I*^2^ = 22%); therefore, the fixed-effects model was used.

*Generation-specific complications*; component fractures occurred in 11 cases out of 1339 hips in the 3rd-generation (0.8%) but decreased significantly to two cases out of 939 hips in the 4th-generation (0.2%) (*p* = 0.057). This was not the case when comparing the prevalence of squeaking, which was 2.9% in the 3rd-generation (40/1339 hips) and 2% in the 3rd-generation (19/939 hips), a statistically insignificant difference (*p* = 0.178).

### 2-Comparison meta-analysis

**Fig. 3:**
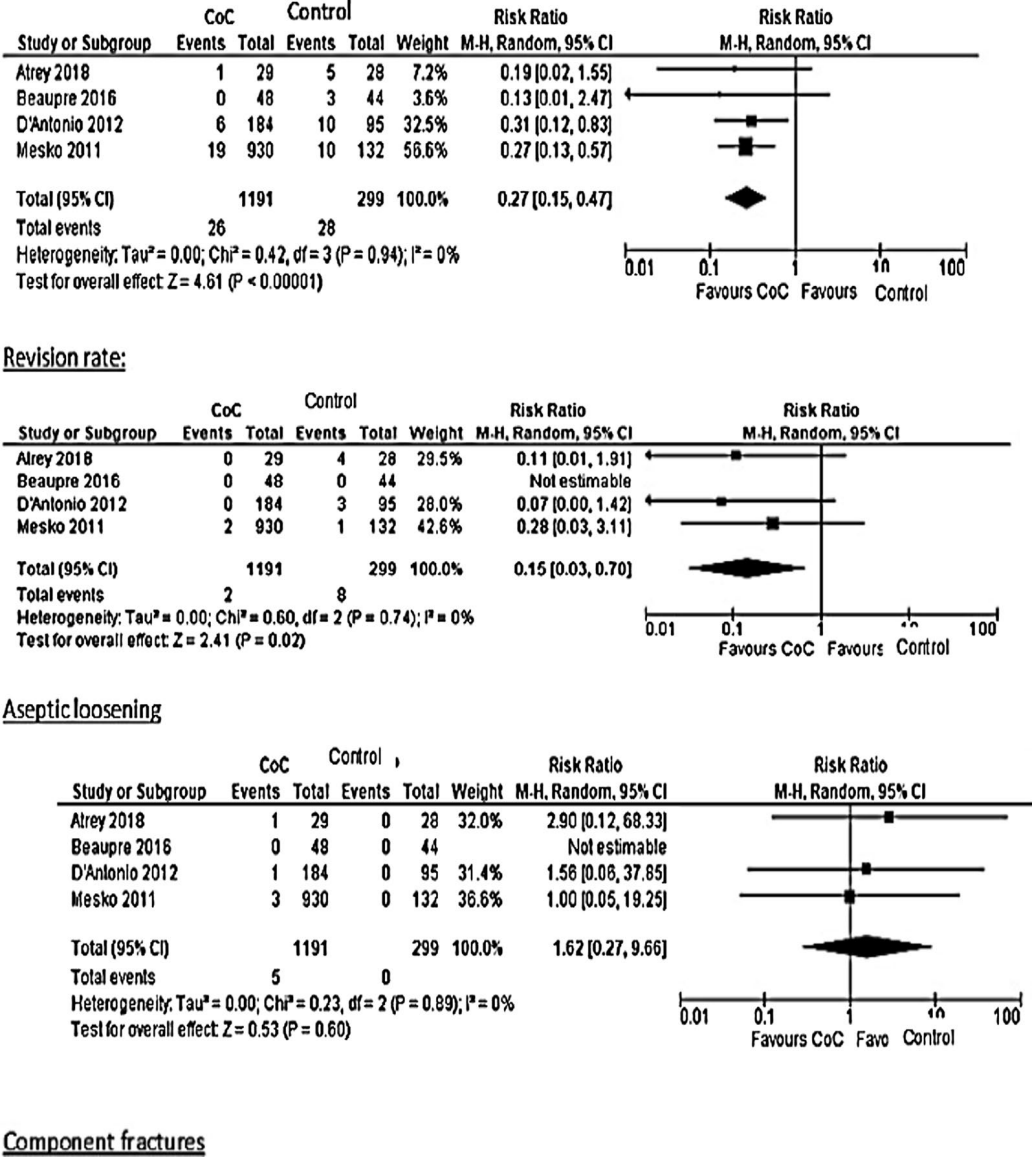
Forest plot of comparison meta-analysis including revision rates, aseptic loosening, and components fracture

Four RCTs compared the outcomes of THA between CoC and polyethylene bearings, including 1191 hips. The survival rate for CoC was 96% (95% CI; 92.8–98.7), and for the polyethylene was 92.4 (96% CI; 85.1–97.4). *Risk Ratio* (RR) of revision due to any cause was 0.27 (95% CI; 0.15–0.47), and for aseptic loosening was 0.15 (95% CI; 0.03–0.70); both were *in* favour of CoC. Contrary to the results mentioned above, RR for component fractures was 1.62 (95% CI; 0.27–9.66), favouring the polyethylene group as is shown in Table 3 and Fig. 3.

**Table 3:** Comparison between CoC and polyethylene

Hips no	Revision	Aseptic loosening	Component fracture	Survival analysis (%)
Atrey 2018				
*CoC (29)	1	0	1	89
**CoP (28)	5	4	0	85
Beaupre				
CoC (48)	0	0	0	94
CoP (44)	3	0	0	90
D'Antonio				
CoC (184)	6	0	1	99.3
***MoP (95)	10	3	0	98.9

*CoC: Ceramic-on-ceramic

**CoP: Ceramic-on-polyethylene

***MoP: Metal-on-polyethylene

### Discussion

Hip replacement surgery is one of the most successful operations in all of medicine. Since the early 1960s, joint replacement surgical techniques and technology improvements have increased total hip replacement effectiveness [24, 25]. According to the Agency for Healthcare Research and Quality, more than 450,000 total hip replacements are performed each year in the United States [26].

Total hip arthroplasty is becoming more common in younger patients around the world. American Academy of Orthopaedic Surgeons (AAOS) reported a 123% increase in THA rates in the 45–64 age group from 2000 to 2009 [27]. Younger patients are more active and have a longer life span, so the option of THA bearing surface may be perplexing for a surgeon, leading to the recommendation of hard-on-hard bearings as CoC over hard-on-soft bearings (CoP or MoP) [1, 28], with suggested survival to be inferior compared to the elderly population [29]. THA implants are only approved for people if they have a 10-year revision rate of less than 5%, according to the UK National Institute of Health and Care Excellence benchmark in 2014 [30].

A decade ago, a meta-analysis tested three bearings CoC, MoP, and metal on metal (MoM), when used in young patients, and concluded that ceramic articulations had the lowest survival among the bearing surfaces (89% 10-year survival) with the highest survival rate for MoM articulations (95.4%) [31]. However, the ceramic tested in that study was the available second-generation before developing the 3rd and 4th ceramic generations. Besides, a substantial body of evidence has developed against MoM implants after the reported higher complication rates and premature failure [32–34].

Ceramic-specific complications include ceramic fracture, squeaking, and aseptic loosening, have been linked to a higher failure of CoC implants than hard-on-soft implants as MoP [35]. Early ceramic fractures rate was reported up to 5% in the 1st and 2nd generations [36], with overall survivorship between 75 and 84% after ten years [37], that improved with the introduction of hot iso-static pressing (HIP) techniques, which reduced the grain size during ceramic manufacturing. HIP improved the wear properties, resistance to fracture, and durability [38]. These techniques allowed the release of 3rd -generation ceramic (Biolox forte) in 1995 and after that the 4th -generation (Biolox delta) in 2000 [8], with a reduction in the bearing fracture rate to be as low as 0.126% for liners and 0.009% for femoral heads [39, 40].

Our meta-analysis found some expected findings consistent with previously published studies, and it also produced some new data by combining the included studies. In the proportional analysis, the 10-year survival rate of CoC THA was recorded as 96% (95% CI; 95.4–96.8, *P* < 0.001), tested in 13 studies that included 2278 patients with the 3rd- and 4th-generation CoC bearings. This survival rate is similar to the patients' of 60 years or more, recorded to be from 95.6 [24] to 96.7 [25]. Only one study had a survival rate of less than 90% [6]; however, this study has the most extended follow-up in the review (15 years) with the smallest number of cases. The ceramic fracture rate was recorded at a rate of 0.62 (95% CI; 0.341–1.034), with an audible squeak rate of 2.687% (95% CI; 1.279–4.593). The rate of aseptic loosening was found to be 0.516 (95% CI; 0.265–0.903).

Sandwich ceramic cup design with intervening polyethylene between the ceramic liner and metal shell had been attributed to a high fracture rate. Kawano et al. reported ceramic sandwich fractures in 50 hips out of 270 with a 13-year survival rate were only 68.0% [41]. One study reported two sandwich ceramic liners fractures out of 90 hips [21]. Another situation to be avoided was the cup edge loading by reducing the acetabular abduction angle and malalignment with subsequent stripe wear reduction. Reduction in the cup edge loading reduces the rate of ceramic fracture [42].

Biomechanically, ceramics offer the best wear resistance, owing to wettability and fluid film formation [43], and have a lower incidence of osteolysis than metal-on-metal, so considered the ideal bearing surface in the younger active patient with a low rate of aseptic loosening [44, 45].

Squeaking for CoC bearing had a reported rate from < 1 to 21%, with presumed multifactorial aetiology [46]. Elevated rim of the acetabular cup and excessive or insufficient cup anteversion cause the neck impingement with squeaks [47]. Patient factors such as younger age, increased height and weight, and rigorous physical activity were also accused, together with the stem design and the type of ceramic (less voice with Delta than Forte types) [48–51]

Due to the 4th-generation ceramics’ increased toughness and burst strength, it was expected that fractures would occur at a much lower rate than the 3rd -generation ceramics' reported fracture rate. The review reported this as having a fracture rate of 0.8%, compared to 0.2% for the 4th -generation. However, no significant difference in squeaking rates was observed. Recent articles directly comparing the 3rd- and 4th- generations reported similar findings in patients with an average age of greater than 50 years. [52, 53]. Similarly, in 2020, Luceri et al. reported excellent outcomes following THA with Biolox Detla implants in patients aged 14–20 years at the time of surgery and followed for an average of 3.3 years (range 0.7–10.1 years) [54].

Comparing CoC to polyethylene, this meta-analysis revealed better CoC bearings outcomes than the results obtained by Shetty et al. [31]. In their study, CoC-bearing surfaces (three studies, sample size of 254 patients) revealed a 10-year survival rate of 88.9% (95% CI; 79.4–95.7%), while MoP liners (20 studies, sample size of 3592 patients) achieved pooled 10-year survival rates of 92.0% (95% CI; 89.4–94.2%). In our review, the 10-year survival (four studies with 1191 hips) was 96% (95% CI; 92.8–98.7). We included only studies after 2006 to find a 10-year follow-up of Forte and so, Delta ceramics. Revision rates and aseptic loosening were in favour of CoC. Comparable results were obtained by Hu et al. [55], in a more recent meta-analysis which tested these rates among 3rd- and 4th -generation ceramic THA. However, this meta-analysis included studies with a follow-up of less than ten years. Bearing surfaces fracture rates fell in disfavour compared to the polyethylene group in all meta-analyses. In our meta-analysis, five ceramic fractures were among 1191 (0.4%), and in Hu et al. study, four fractures were recorded among 601 (0.66).

All studies in this systematic review have high methodological quality, and all RCTs included in the meta-analysis have follow-ups more than ten years with patients less than 60 years of age (average 44). The study strengths are the large patient sample size (2278 hips) and the ability to examine multiple risk factors. Besides, this study was able to combine multiple implants from various manufacturers. Finally, all the studies were published in the last fifteen years.

One weakness is that most of the included studies were level-of-evidence 2–4 studies. Only four of the included 13 studies were level-1-study. An effort was made to perform a "best evidence meta-analysis," including studies with appropriate methodology. Not every study examined the same variables. Thus, for each of the subgroup analyses, there were a different number of studies included.

## Conclusion

Ceramic on ceramic-bearings in current THA has been associated with improved 10-year results and survival when used in patients less than 60 years than what was documented before. The improved manufacturing process of ceramic surfaces has a significant impact on these results.

## Data Availability

Available on request.

## Abbreviations

THA: Total hip arthroplasty
CoC: Ceramic-on-ceramic
RCTS: Randomised controlled trials
PRISMA: Preferred reporting items for systematic reviews and meta-analyses statement
MoP: Metal on polyethylene
MoM: Metal on metal
HIP: Hot iso-static pressing
